# Analyzing Spatio-Temporal Dynamics of Grassland Resilience and Influencing Factors in the West Songnen Plain, China, for Eco-Restoration

**DOI:** 10.3390/plants13131860

**Published:** 2024-07-05

**Authors:** Gefei Wang, Zhenyu Shi, Huiqing Wen, Yansu Bo, Haoming Li, Xiaoyan Li

**Affiliations:** 1 College of Earth Sciences, Jilin University, Changchun 130061, China; 2 Juungar Banner Natural Resources Bureau, Ordos 010300, China

**Keywords:** salt-alkali soil, grassland ecosystem, resilience, spatio-temporal variation, the west Songnen Plain, eco-restoration

## Abstract

Grassland plays an indispensable role in the stability and development of terrestrial ecosystems. Quantitatively assessing grassland resilience is of great significance for conducting research on grassland ecosystems. However, the quantitative measurement of resilience is difficult, and research on the spatio-temporal variation of grassland resilience remains incomplete. Utilizing the Global Land Surface Satellite (GLASS) leaf area index (LAI) product derived from MODIS remote sensing data, along with land cover and meteorological data, this paper constructed the grassland resilience index (GRI) in the west Songnen Plain, China, a typical region with salt and alkali soils. This paper analyzed the spatio-temporal changes of the GRI and explored the contribution of climate factors, human activities, and geographical factors to the GRI. The results revealed that from 2000 to 2021, the GRI in the study area ranged from 0.1 to 0.22, with a multi-year average of 0.14. The average GRI exhibited a pattern of high-value aggregations in the north and low-value distributions in the south. Trend analysis indicated that areas with an improved GRI accounted for 59.09% of the total grassland area, but there were still some areas with serious degradation. From 2000 to 2015, the latitude and mean annual temperature (MAT) were principal factors to control the distribution of the GRI. In 2020, the mean annual precipitation (MAP) and MAT played important roles in the distribution of the GRI. From 2000 to 2021, the influence of human activities was consistently less significant compared to geographical location and climate variables.

## 1. Introduction

Grassland ecosystems are one of the significant foundations for maintaining the sustainable development of the whole human society. In China, grassland is the largest terrestrial ecosystem in the area [1]. However, due to the combined impacts of climate change and human activities, nearly 49% of the world’s grassland areas have undergone varying degrees of degradation [2], with nearly 70% to 90% of the grassland area in China also undergoing degradation to varying extents [3,4,5]. The intensification of human activities and global climate change has led to varying degrees of damage to grassland ecosystems and exacerbated soil salinization and sodication, particularly in arid and semi-arid regions. This has significantly impaired the services of grassland ecosystems and has had a profound impact on human society’s production and livelihoods [6,7]. The sustainable utilization of ecosystems faces enormous challenges [8]. However, resilient grasslands ensure the continued provision of vital ecosystem services, such as carbon sequestration, water regulation, soil fertility, biodiversity conservation, and so on [9].

Grassland resilience refers to the capacity of grassland ecosystems to resist or recover from disturbances, returning to their original state before the stress occurred [10,11]. A system’s resilience enhances its stability. Environmental heterogeneity causes variations in grassland resilience. Excessive human or climatic stress beyond the grassland’s recovery capacity leads to its degradation [12,13]. Therefore, quantifying ecosystem resilience is essential for assessing degradation risks and promoting sustainable grassland management.

Previous studies have indicated that grassland resilience is closely linked to the number of dominant species within a native community, grassland coverage, and land surface environmental conditions [14]. Most of the time, these factors are obtained through on-site measurements, a method that, despite its accuracy, is labor-intensive and limited by geographical scopes [15]. The seasonal occurrence of plants, the accessibility of field locations, and the experience of observers can also affect measurement results [16].

Considering the limitations of in-situ measurements, remote sensing technology has been extensively employed to assess grassland vegetation type, growth, and grass yield on a regional scale [17,18]. Remote sensing can be used to monitor and quantify different phenomena on the Earth’s surface at different spatial and temporal resolutions on a large scale [19]. Some studies employ remote sensing data to directly identify vegetation anomalies for evaluating terrestrial ecosystem resilience, such as the normalized difference vegetation index (NDVI) [20]. Harris et al. utilized the duration of vegetation recovery from disturbances as an indicator of ecosystem resilience [21]. Seddon et al. devised the vegetation sensitivity index to monitor alterations in the resilience of terrestrial ecosystems [22]. Based on the critical deceleration theory, Verbesselt et al. used the temporal autocorrelation of vegetation to measure the resilience of tropical forests [23]. Although these studies have performed quantitative measurements of ecosystem resilience at regional levels using different indices, the results are multi-year averages and cannot describe changes in resilience or potential causes. Wu et al. used the ratio of the maximum pressure that the ecosystem can recover from to the time it takes for the ecosystem to recover from the maximum pressure as a proxy for measuring terrestrial ecosystem resilience [24]. Not only are the index changes represented by vegetation considered, but also the time characteristics of resilience are measured, which essentially reflects the strength of resilience.

This research utilized the LAI as a measure of grassland resilience, a departure from traditional metrics such as gross primary productivity, biomass, or NDVI, which have been widely applied in previous studies to construct statistical models [24,25,26]. The LAI is an essential vegetation parameter that characterizes canopy structure and leaf density [27]. The LAI is one of the important indicators of the light utilization efficiency of plants, which can reflect the photosynthesis rate of plants. By monitoring the LAI, the photosynthetic rate of grasslands can be evaluated to predict the yield and quality of grasslands. In comparison, the LAI is estimated directly from remote sensing data through physical models, which not only has high accuracy but also enables analysis over extended periods [24,28,29].

The Songnen Plain is the largest saline-sodic region in China [30,31,32]. During the period 1954–2000, human activities such as overgrazing led to a reduction in grassland area in the western Songnen Plain, while land affected by soil salinization and sodication increased [33]. Since 2000, a series of policies have been implemented, including the Grassland Ecological Subsidy and Award Scheme (GESAS) [34] and the grazing exit plan [35]. The implementation of these policies has played various roles in promoting the restoration of grassland vegetation and enhancing grassland productivity and resilience in different regions [36]. In addition, the study area belongs to a high-latitude region that is particularly sensitive to climate change [37]. Under the joint effects of human activity and climate change, the assessment of grassland restoration effects has become one of the focal issues. Grassland resilience is the most direct indicator of ecosystem health and a prerequisite for restoration effectiveness, which is of paramount importance for implementing effective grassland management, ensuring ecological health, and regional sustainable development.

This study constructs a grassland resilience index based on time series LAI data. Combined with a variety of time series analysis models and geographic detector models, the spatio-temporal trend of the GRI and its driving factors in the study area from 2000 to 2021 were analyzed. Our objectives are to (1) analyze the spatio-temporal characteristics of grassland resilience over a long time series in the west Songnen Plain, (2) explore the trend characteristics of grassland resilience, and (3) investigate the effects of environmental and anthropogenic factors on grassland resilience.

## 2. Materials and Methods

### 2.1. Study Area

**Figure 1:**
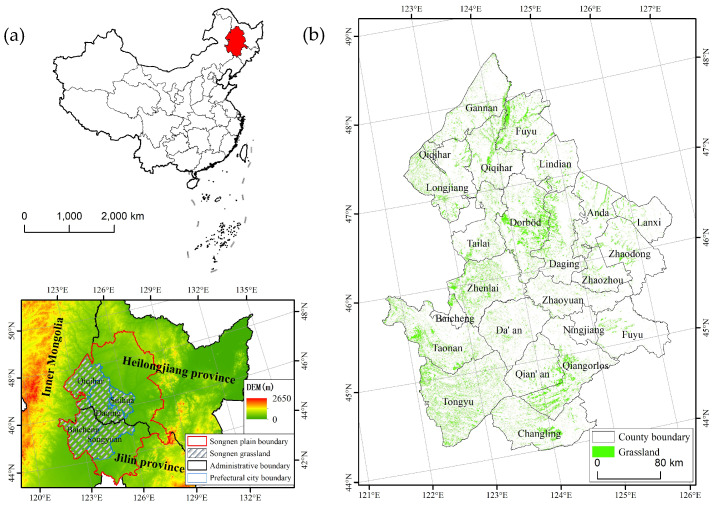
The location of the study area (**a**) and the distribution of grasslands (**b**).

The study area is situated in the western part of the Songnen Plain, northeast China (Figure 1). It is situated between 121°36′ E to 126°36′ E and 44°00′ N to 48°35′ N across 23 counties in the Heilongjiang and Jilin provinces, covering over 101.51 × 10^3^ km^2^. The terrain of the study area is dominated by low plains formed by river alluvial deposits, and the altitude ranges from 200 to 300 m. The study area is characterized by a semi-arid and semi-humid climate, with an average temperature ranging between 4 °C and 6 °C and a mean annual precipitation (MAP) varying from 300 mm to 650 mm. Under conditions of moderate rainfall and a suitable climate, a meadow system with *Leymus chinensis* as the dominant species was developed. Zonal soil types from east to west are brown forest soil (Haplic Luvisol, FAO), black soil (Luvic Phaeozem, FAO), chernozem (Haplic Chernozems, FAO), and chestnut soil (Haplic Kastanozem, FAO), among which black soil and chernozem constitute 45.6% of the total land area [38]. The study area encompasses grasslands whose land cover types remained consistent from 2000 to 2021.

### 2.2. Data Sources

#### 2.2.1. Land Cover Data

Land cover data is based on the annual land cover dataset of China from 2000 to 2021, interpreted from Landsat images with a spatial resolution of 30 m (https://zenodo.org/record/5816591). Land cover types were classified into cropland, built-up land, grassland, woodland, wetland, water body, and barren land in this dataset. Python 3.11.0 was used to project, clip, and mosaic the land cover data in the study area for 22 years, and grassland was extracted. This dataset has an overall accuracy of 79.31%.

#### 2.2.2. Remote Sensing Image Data

The remote sensing images utilized in the study are GLASS LAI products in the growing season from May to September 2000–2021 (http://www.glass.umd.edu/Download.html), with a temporal resolution of 8 days and a spatial resolution of 500 m. According to the geographical location of the study area, the data row number is h26v04, and the LAI data from the 121st to 273rd days from 2000 to 2021 were downloaded, with 20 scenes per year and 440 scenes in total. Python was used for batch processing of format conversion and projection transformation. The preprocessed data was used to construct the GRI.

#### 2.2.3. Climate Data

The climate data included mean annual temperature (MAT) and MAP. The monthly average temperature and precipitation datasets with a spatial resolution of 1 km from 1901 to 2021 were derived from the website of the National Earth System Science Data Center (http://www.geodata.cn). The data is of the INT16 type and stored in a network common data form (NDTCDF, NC). Python was used for the batch processing of extractions and calculations. The dataset is based on the National Centers for Environmental Prediction/National Center for Atmospheric Research (NCEP/NCAR) global near-surface monthly average temperature data, combined with the quantitative relationship between the digital elevation model (DEM), NDVI, and geographical location. A point-to-kilometer statistical downscaling regression tree model was established. The accuracy verification results showed an R^2^ range from 0.865 to 0.95, and the root mean square error (RMSE) ranged from 1.88 °C to 2.68 °C [39].

#### 2.2.4. Socio-Economic Data

Human activity data mainly include gross domestic product (GDP) data and population density (PD) data. National GDP data with a spatial resolution of 1 km in 2000, 2005, 2010, 2015, and 2020 were derived from the resource and environmental science data registration and publishing system (http://www.resdc.cn/DOI). The PD data at the same time span were obtained from the World POP dataset with a spatial resolution of 1 km (https://www.worldpop.org/). Both GDP and PD data in the study area were extracted based on the boundary of the research area.

### 2.3. Methods

#### 2.3.1. Construction of GRI

**Figure 2:**
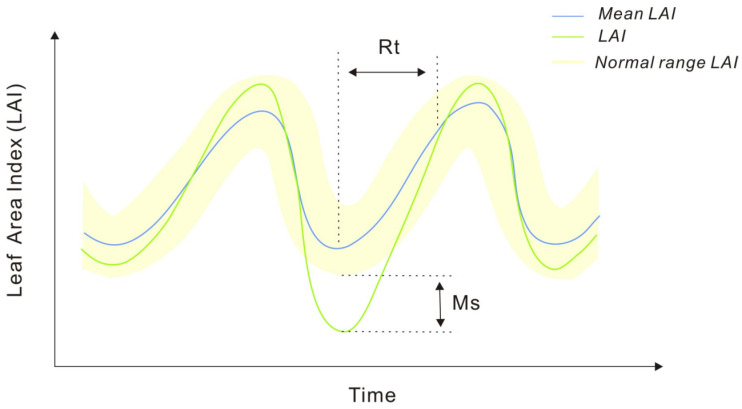
Simplified process of ecosystem anomaly [24].

The LAI data were chosen as the proxy of vegetation to present the GRI owing to its reliable accuracy in vegetation monitoring. Referring to Wu et al. [24], the GRI was constructed by considering the LAI changes and recovery time simultaneously. The normal range of the LAI was determined by the mean and standard deviation. When the LAI is lower than the normal range, the difference between it and the normal range is the maximum pressure that the ecosystem can recover from (Ms). The time of the Ms appearance marks the beginning of the recovery process, while the time taken to return to the normal range signifies the end of recovery. The duration representing the ecosystem’s recovery from the maximum pressure is denoted as Rt (Figure 2).

The GRI was defined as a ratio of Ms to Rt:

(1) $$\mathrm{Ms}={MEAN}_{j}-{\mathrm{LAI}}_{\mathrm{ij}\left(\mathrm{lowest}\right)}-\beta \times {\mathrm{STD}}_{j}$$

(2) GRI=Ms/Rt

where *Ms* is the maximum stress that grasslands can withstand and recover from, ${MEAN}_{j}$ is the average value of the LAI in the month j in the research period span, ${LAI}_{ij(lowest)}$ is the lowest value of the LAI in the month j and year i in the abnormal period, β is the parameter used to adjust the normal period, and ${STD}_{j}$ is the standard deviation of the LAI in the month j in the research period.

Because the occurrence time of anomalies is not fixed and the duration is different, it is necessary to set up the rules for calculating the GRI: when there are no abnormalities in the current year, the GRI is set to be equal to the resilience of the previous year; when the recovery time crossed more than one year, the GRI value is set to be equal to the resilience at the beginning of the recovery period; when there are multiple anomalies in the current year, the GRI is calculated by averaging the GRI values detected throughout the current year.

The annual GRI was normalized by the formula as follows:

(3) $${GRI}_{new}=\frac{x-Min}{Max-Min}$$

where *x* is the quasi-normalized data of *GRI*, ${GRI}_{new}$ is the normalized data of GRI, and Max and Min are the maximum and minimum values in the current *GRI* data, respectively.

#### 2.3.2. Trend Analysis for GRI

The Theil–Sen median trend analysis and Mann–Kendall (M–K) test methods were used to study trend characteristics of the GRI in the west Songnen Plain from 2000 to 2021 [40,41,42].

Based on the Theil–Sen Median trend analysis method, this study calculated the change rate (*Slope*) of each grid by pixel in the time series of the GRI. This approach reflects the comprehensive trend of changes in regional resilience during the study period.

(4) $$slope=Median\left(\frac{{GRI}_{j}-{GRI}_{i}}{j-i}\right)(2000≦i≦j≦2021)$$

where ${GRI}_{j}$ and ${GRI}_{i}$ denote the pixel *i*-year and the pixel *j*-year of the GRI, respectively. When *Slope* > 0, it shows that the GRI of this pixel has changed positively in the past 22 years; that is, the annual average GRI has increased. When *Slope* < 0, it shows that the GRI of this pixel has changed negatively in the past 22 years; that is, the average annual GRI has decreased. A trend value of 0 indicates that the GRI of this pixel has not changed.

The M–K nonparametric statistical test method was employed to assess the significance of the change trend of GRI. This study used raster data overlay analysis to superimpose the results of the change trend of the GRI in the west Songnen Plain with the results of the M–K test.

For the time series variable x of $x_{1},x_{2}$,…$x_{n}$, where x denotes the year-by-year resilience of each pixel, the trend test is conducted using the test statistic *Z*. The *Z* value is calculated as follows:

(5) $$S=\sum_{i}^{n-1}\sum_{j=i+1}^{n}sgn\left(x_{i}-x_{j}\right)$$

(6) $$sgn(\theta )=\left\{\begin{array}{c} 1,\theta >0\\ 0,\theta =0\\ -1,\theta <0 \end{array}\right.$$

(7) $$Var\left(S\right)=\frac{n(n-1)(2n+5)}{18}$$

(8) $$Z=\left\{\begin{array}{c} \frac{S}{\sqrt{Var\left(S\right)}}(S>0)\\ 0(S=0)\\ \frac{S+1}{\sqrt{Var\left(S\right)}}(S<0) \end{array}\right.$$

where sgn is a sign function, *θ* represents the value of (*x_i_* − *x_j_*), and *n* is the amount of data in the time series. Under the assumption of a two-sided trend test, when $Z>U_{1-\alpha /2}$, the null hypothesis is rejected, and the trend is considered significant at the given significance level α of 0.05. Based on previous studies [43,44,45,46,47] and considering the GRI characteristics in the study area, the change trend of resilience was categorized into six grades (Table 1). When *slope* > 0 and *Z* ≥ 2.58, *Z* ≥ 1.96, it indicated that the trend had exceeded the confidence level of 99% and 95% significance test, which was defined as an extremely significant and significant improvement, respectively. When *Z* < 1.96, it indicated that the trend was a modest improvement. When *slope* < 0 and *Z* ≥ 2.58, *Z* ≥ 1.96, it was defined as an extremely significant and significant degradation, respectively. When *Z* < 1.96, it indicated that the trend was a modest degradation.

#### 2.3.3. Simple Linear Regression Model

A simple linear regression model is used to describe the trend changes of climate elements in the west Songnen Plain, specifically:

(9) $$Slope=\frac{N\times \sum_{i=1}^{N}i\times x_{i}-\left(\sum_{i=1}^{N}i\right)\left(\sum_{i=1}^{N}x_{i}\right)}{N\times \sum_{i=1}^{N}i^{2}-{\left(\sum_{i=1}^{N}i\right)}^{2}}$$

where *Slope* is the slope of the regression equation for each pixel, *N* is the length of the time series data (*N* = 22), and *x_i_* is the average climate data for the *i*-th year. If the *Slope* > 0, it indicates an increase in the climate data values. If *Slope* < 0, it indicates a decrease in the climate data values.

#### 2.3.4. Pearson Correlation Analysis Method

To quantify the correlation between grassland resilience and climate factors, this study employed the Pearson correlation analysis method to quantitatively assess the relative contribution rates of the annual average temperature and annual precipitation to changes in grassland resilience [48]. The calculation formula is as follows:

(10) $$r_{xy}=\frac{\sum_{i=1}^{n}\left[\left(x_{i}-\bar{x}\right)\left(y_{i}-\bar{y}\right)\right]}{\sqrt{\sum_{i=1}^{n}{\left(x_{i}-\bar{x}\right)}^{2}}\sqrt{\sum_{i=1}^{n}{\left(y_{i}-\bar{y}\right)}^{2}}}$$

where *r_xy_* is the correlation coefficient between variables *x* and *y*, *x_i_* represents the grassland resilience in year *i*, *y_i_* represents the annual average temperature (or annual precipitation) in year *i*, $\bar{x}$ is the mean grassland resilience, and $\bar{y}$ is the mean annual average temperature (or annual precipitation).

The value of *r_xy_* ranges between −1 and 1. When *r_xy_* > 0, it indicates a positive correlation between the variables *x* and *y*; when *r_xy_* < 0, it indicates a negative correlation. The larger the absolute value of *r_xy_*, the stronger the correlation between the variables *x* and *y*.

#### 2.3.5. Geo-Detector Model

In this paper, the differentiation and factor detector in the geographic detector was introduced to quantitatively analyze the intensity of the factors affecting the spatial differentiation of the GRI [49,50,51,52]. The expression is as follows:

(11) $$q=1-\frac{1}{n\sigma^{2}}\sum_{i=1}^{m}n_{i}\times \sigma^{2}$$

where *q* is the driving factor explanatory power of GRI, *n* and $\sigma^{2}$ are the sample size and variance of the study area, respectively, and *i* = 1, 2…, *m* represents the classification of influence factors. The range of the *q* value is between 0 and 1, where a larger *q* value indicates a stronger explanatory power of the factor on the spatial distribution of the GRI.

The relative contribution rate (RCR) is used to quantify the contribution of individual components to a total measure. The formula for RCR is given as follows:

(12) $${RCR}_{i}=\frac{q_{i}}{\sum_{j=1}^{n}q_{j}}\times 100\%$$

where *RCR_i_* is the relative contribution rate of the *i*-th component, and *q_i_* represents the value of the *i*-th component or factor being considered in the calculation.

**Figure 3:**
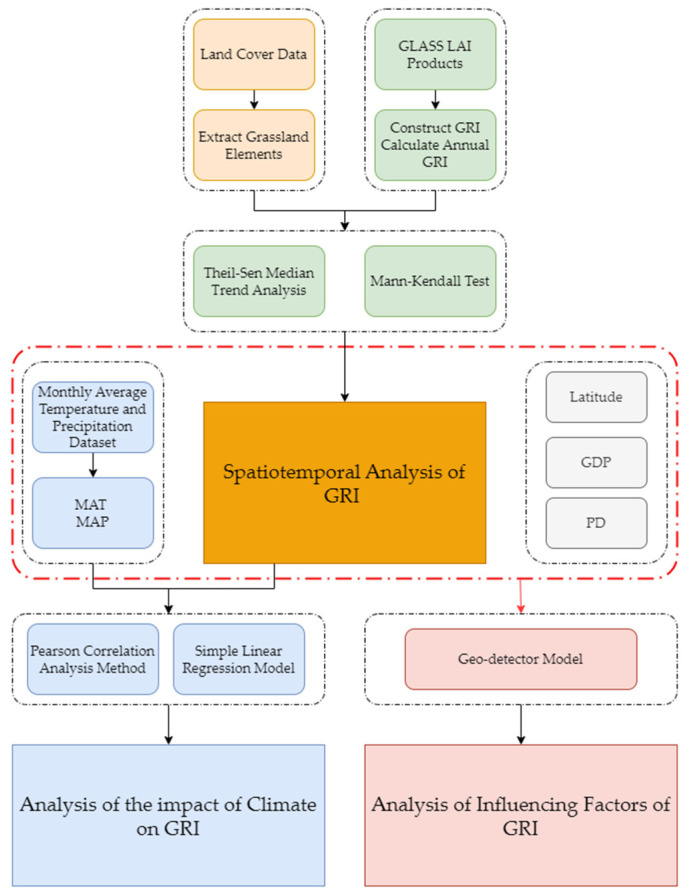
Technical roadmap.

The technical workflow presented in this study is illustrated in the following figure (Figure 3).

## 3. Results

### 3.1. Spatio-Temporal Characteristics of GRI

#### 3.1.1. Temporal Characteristics of GRI

**Figure 4:**
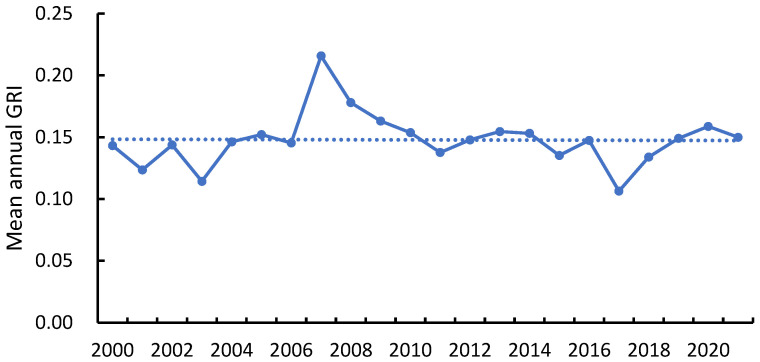
Variation of the mean annual GRI in the west Songnen Plain from 2000 to 2021.

From 2000 to 2021, the annual average GRI in the west Songnen Plain varied between 0.10 and 0.22, with a multi-year average value of 0.14 (Figure 4). The trend curve shows fluctuating dynamics, but the overall trend remained relatively stable. The GRI in the study area increased from 0.14 in 2000 to 0.15 in 2021, indicating an overall growth rate of 7.14% over 22 years and an average annual growth rate of 0.32%. The maximum GRI was 0.22 in 2007, and the minimum GRI was 0.11 in 2017.

#### 3.1.2. Spatial Distribution and Variation Characteristics of GRI

**Figure 5:**
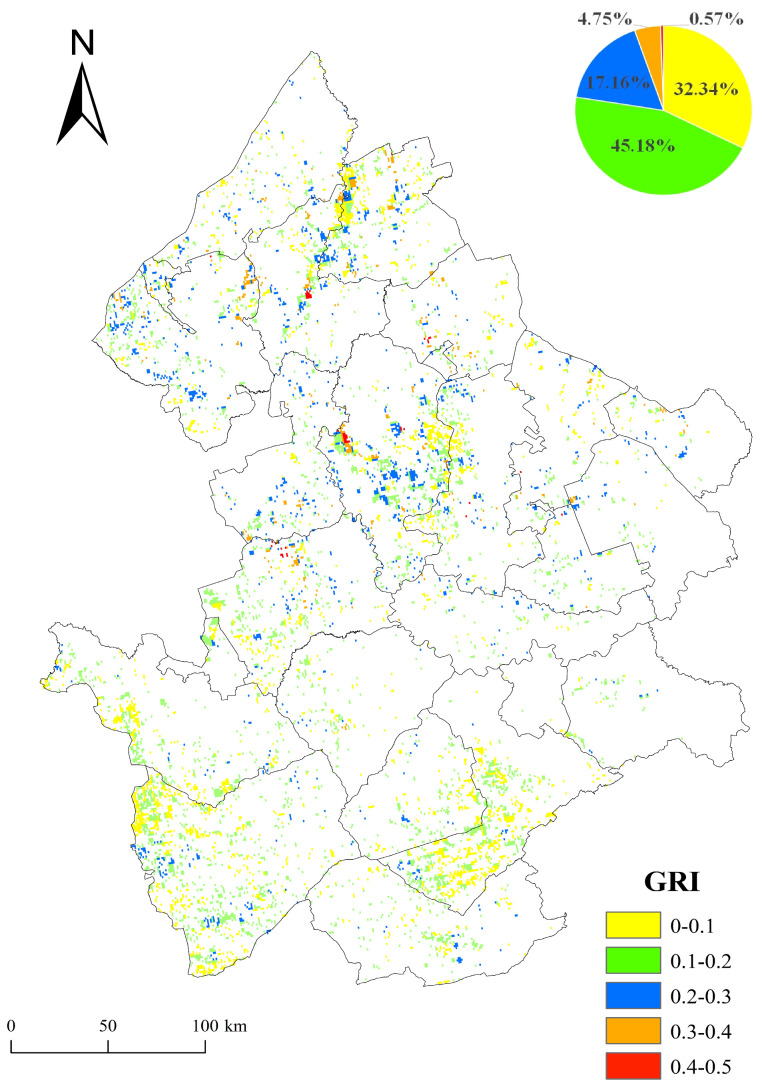
Spatial distribution of the mean GRI in the west Songnen Plain from 2000 to 2021.

The average resilience of grasslands from 2000 to 2021 in the study area was calculated and divided into five grades (Figure 5). The results showed that the spatial heterogeneity of the GRI in the west Songnen Plain was obvious. High GRI values were aggregated in the north, and the low GRI values were distributed in the south. The mean GRI in the Heilongjiang Province was higher than that of the Jilin Province. The higher values of the GRI between 0.4 and 0.5 in the Heilongjiang Province were concentrated in Anda, Daqing, Durbert, Lindian, Longjiang, Qiqihar, and Zhaozhou, while those in Jilin Province were only sporadically distributed in Zhenlai. Grasslands with GRI values between 0 and 0.2 accounted for 77.52%, indicating a relatively low overall resilience during the past 22 years. 

### 3.2. Trend Analysis of GRI

**Figure 6:**
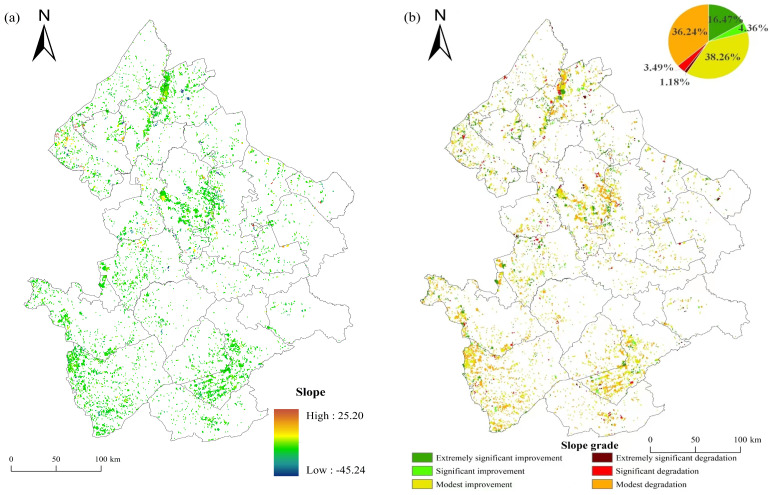
Spatial distribution (**a**) and grade (**b**) of the slope for the GRI from 2000 to 2021.

Based on the Theil–Sen Median trend analysis method, the slope of the GRI was calculated (Figure 6a) and classified into six grades (Figure 6b). The slope of the GRI varied between −45.23 and 25.20, and the mean slope of the GRI in the study area was 0.15. Grasslands with extremely significant improvement were mainly distributed in the Heilongjiang Province.

From 2000 to 2021, the area of grassland with the improved GRI was 5390 km^2^, accounting for 59.09% of the total grassland area. The grassland with a modest improvement in resilience was the largest, with an area of 3490 km^2^ and accounting for 38.26% of the total grassland area. The area of grassland with significant and extremely significant improvement in resilience was 398 km^2^ and 1502 km^2^, respectively, representing 4.36% and 16.47% of the total grassland area, respectively. The grassland with an area of 3305 km^2^ was characterized by a moderate degradation of resilience, accounting for 36.24% of the total grassland. The grassland with significant degeneration was only 318 km^2^, accounting for 3.49% of the total grassland. Grasslands with an extremely significantly decreased resilience were distributed sporadically and only accounted for 1.18%, primarily concentrated in the north of the study area.

### 3.3. Analysis of the Impact of Climate on GRI

**Figure 7:**
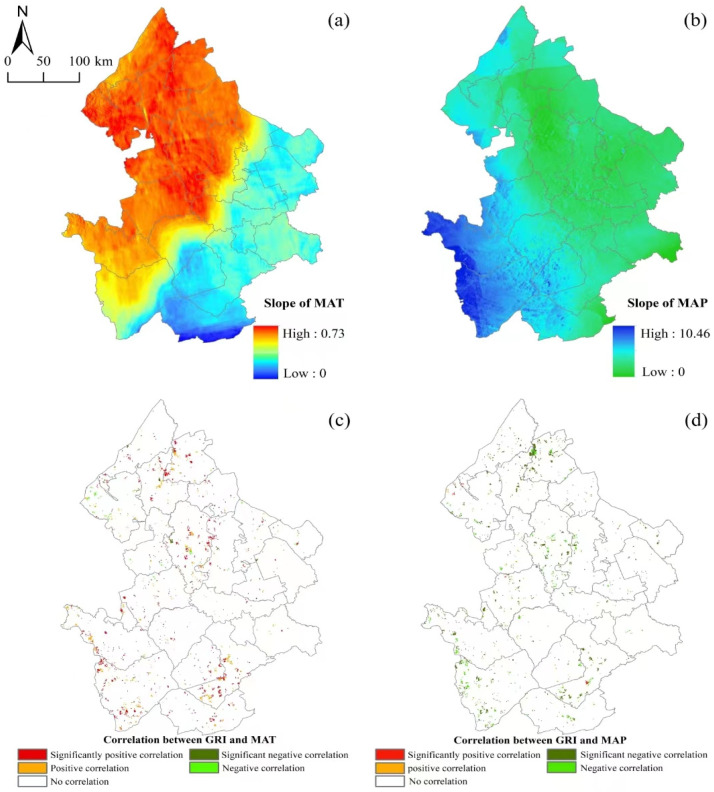
Slope of the MAT (**a**) and MAP (**b**); Spatial distribution of the correlation between the MAT (**c**), MAP (**d**), and GRI.

The results of the trends in the MAT (Figure 7a) and MAP (Figure 7b) in the western Songnen Plain over the 22 years from 2000 to 2021 show that the MAT exhibited an overall increasing trend, with the rate of increase gradually intensifying from southeast to northwest. Similarly, the MAP showed an overall increasing trend, with the growth rate gradually intensifying from east to west.

The correlation coefficient between the GRI and the multi-year average temperature in the western Songnen Plain ranged from −0.78 to 0.91. Areas where the MAT and the GRI exhibited a significant negative correlation (*p* < 0.05) and a negative correlation (*p* < 0.1) accounted for 1.59% and 1.48% of the total study area, respectively. Conversely, areas with a positive correlation (*p* < 0.1) and a significant positive correlation (*p* < 0.05) accounted for 8.40% and 14.97% of the total study area, respectively (Figure 7c). 

The correlation coefficient between the GRI and the multi-year average precipitation ranged from −0.88 to 0.56. Areas where the MAP and the GRI exhibited a positive correlation (*p* < 0.1) and a significant positive correlation (*p* < 0.05) accounted for 0.32% and 0.54% of the total study area, respectively. Conversely, areas with a significant negative correlation (*p* < 0.05) and a negative correlation (*p* < 0.1) accounted for 14.66% and 9.23% of the total study area, respectively (Figure 7d).

### 3.4. Impact of Influencing Factors on GRI

Based on the geo-detector model, the effects of different influencing factors on the GRI in the study area in 2000, 2005, 2010, 2015, and 2020 were compared. It was found that there were significant differences in the effects of the same factor on the GRI at different scales.

#### 3.4.1. RCR of Influencing Factors to the Spatial Distribution of GRI at Regional Scales

**Figure 8:**
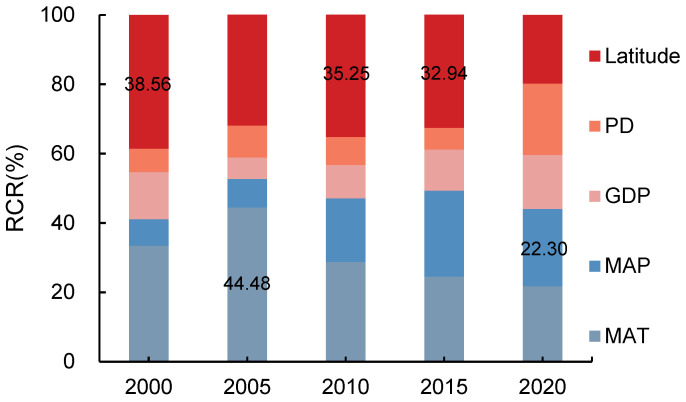
The RCRs of the influencing factors to the spatial distribution of the GRI from 2000 to 2021.

The RCRs of factors showed significant differences between 2000 and 2015 but became more uniform in 2020 (Figure 8). From 2000 to 2015, the latitude and MAT were principal factors that controlled the distribution of the GRI. The RCR of the latitude was 37.73%, 35.25%, and 32.94%, respectively, in 2000, 2010, and 2015, ranking as first in the RCR sequence; the MAT mostly contributed in 2005 with an RCR of 44.48%. In 2020, the MAP and MAT played important roles in the distribution of the GRI, with RCRs of 22.30% and 21.73%, respectively. From 2000 to 2020, the impact of human activities was smaller than that of the geographical location and climate variables. The RCRs of the GDP decreased in 2005 and increased slightly from 2005 to 2020, while that of the PD decreased from 2005 and increased in 2020. The PD contributed minimally in 2000, 2010, and 2015, and GDP had a minimum RCR in 2005 and 2020. The RCR of the PD was less than 10% between 2000 and 2015 but increased to 20.60% by 2020.

#### 3.4.2. RCR of Factors to Dynamic Changes of GRI at Prefecture Level

**Figure 9:**
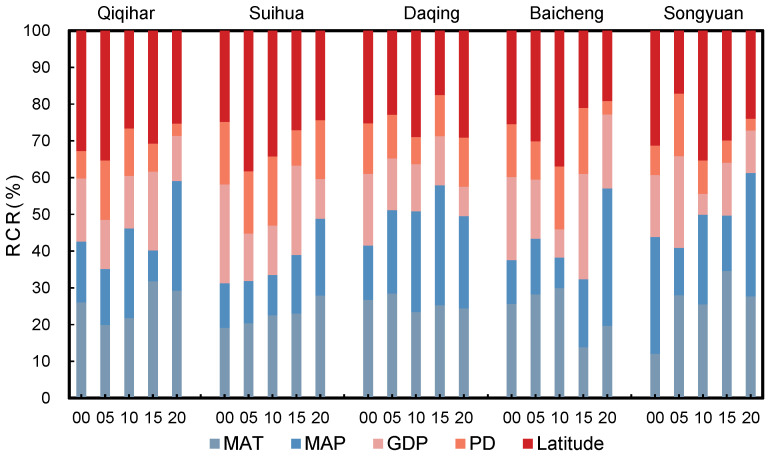
RCRs of factors to the spatial distribution of the GRI in five prefecture-level cities from 2000 to 2020.

Over the past 22 years, latitude has been the main influencing factor on the resilience of grassland ecosystems in Qiqihar and Suihua, with RCRs ranging from a minimum of 24.78% to a maximum of 38.22%, the average mean RCR up to 30%, and then followed by the MAT and MAP with an average RCR of 25.77% and 22.63%, respectively. For Daqing, the RCRs of factors had relatively less variation in different periods. The latitude, MAT, and MAP contributed mostly, and the mean RCR was 28.39%, 25.70%, and 24.51%, respectively. For Baicheng and Songyuan, the latitude still had a maximum RCR, but the mean value dropped to 27.98% and 27.89%, respectively, followed by the MAT with an RCR of 23.49% and 24.60%, respectively. The MAP varied significantly in Baicheng and Songyuan from 2000 to 2021. The MAP of Baicheng had a maximum RCR of 37.41% in 2021 and a minimum RCR of 11.9% in 2000; that of Songyuan was 33.62% in 2021 and 12.88% in 2005. The GDP and PD in five cities made the least contribution from 2000 to 2021, but the RCR of the GDP was higher than that of the PD (Figure 9).

**Figure 10:**
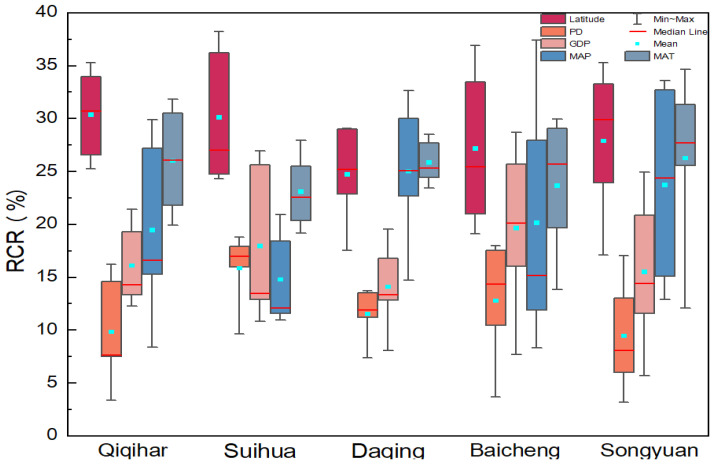
Clustered box plot of RCRs for factors in five prefecture-level cities from 2000 to 2020.

According to the clustered box plot (Figure 10), the maximum explanatory power of the latitude was relatively high in all cities, and the minimum explanatory power of the PD was the lowest, except for Suihua. The RCR of the MAP in Qiqihar, Baicheng, and Songyuan varied over a large range, and the variation range of the RCR for latitude and GDP in Suihua and Baicheng also showed a relatively wide variation.

## 4. Discussions

### 4.1. Spatio-Temporal Characteristics of Grassland Resilience

This study indicated a generally low grassland resilience across the west Songnen Plain, with a multi-year average value of 0.14. Despite significant fluctuations over the past two decades, the overall trend in resilience remained relatively stable. In 2003, there was a relatively small value of the GRI, and in 2007, the GRI reached its peak. This might be attributed to the extensive and extreme droughts that occurred in northern China from 2000 to 2007, particularly the severe drought experienced in the Songnen Plain in 2006 [53]. In 2017, the resilience reached its lowest point, which might be due to the flooding disaster that occurred that year [36,54].

The results of grassland degradation in the west Songnen Plain showed that the average degree of grassland degradation in the Jilin Province was generally more serious than that in the Heilongjiang Province [55]. However, from the perspective of the degradation trend, the area of serious grassland degradation in the Heilongjiang Province was significantly larger than that in the Jilin Province from 2000 to 2020, and grassland degradation in the Jilin Province was mostly moderate. The study by Man et al. also showed the grassland in the Heilongjiang Province changed more dramatically in the past 20 years, and the stability of the grassland ecosystem was poor, while the grassland ecosystem in the Jilin Province was relatively stable [56]. The results of this study showed that high values of the GRI were concentrated in the north and middle of the study area, and low values were distributed in the south. The overall grassland restoration capacity in the Heilongjiang Province was higher than that of the Jilin Province. It is consistent with the above research results in space.

### 4.2. Trend Analysis of GRI

From 2000 to 2021, coverage and net primary productivity (ANPP) in grasslands in the west Songnen plain showed an overall increasing trend [55,57,58]. In the trend analysis of the GRI, we found that 59.09% of grasslands showed an improvement trend, while 40.91% showed a decreasing trend in their resilience, and the degree of improvement was higher than the degree of degradation during the past 22 years. The significantly improved areas were 16.47%, and only 1.08% of the areas showed significant degradation. Moderate improvement and degradation were the main types of trend changes, accounting for 38.26% and 36.24% of the total grassland area, respectively. The overall trend of the GRI in the west Songnen Plain was generally stable and slightly improved. In the related studies [55,59,60], the area of soil salinization and sodication and the degree of land degradation in the entire Songnen Plain in 2000–2020 generally showed a downward trend. The above research results, on the other hand, indicate that the GRI has improved in these years. Furthermore, certain studies have indicated that while the extent of soil salinization and sodication in the west Songnen Plain decreased from 2000 to 2016, the area of grassland salinization and sodication increased by 0.13 × 10^6^ hm^2^ [60]. This discrepancy could be attributed to variations in the time frame considered and the delineation of grassland boundaries.

The research on the ecologically fragile zone in the Songnen Plain revealed that the area of extreme and severe ecological vulnerability showed a downward trend from 2000 to 2020, while that of moderate vulnerability, mild vulnerability, and slight vulnerability showed an upward trend [57]. Among them, the extreme vulnerability and severe vulnerability of Qiqihar, Suihua, Songyuan, and Daqing were greatly reduced to moderate vulnerability. In this study, the areas with a significant increase in the GRI were mainly distributed in Qiqihar, Longjiang, Dorbot, Tailai, Zhenlai, Daqing, Anda, and Lanxi. Changes in reduced vulnerability and improved resilience were basically consistent in spatial distributions, which, to some extent, confirms the reliability and robustness of using the LAI as a proxy for grassland ecosystem resilience.

Although the comprehensive GRI and GRI trend analysis results, along with related research, indicated that the degree of salinization in the west Songnen Plain improved over the past 22 years, severe grassland degradation and increased salinization persisted in some local areas, particularly in the southern part of the study area. Saline-alkali land treatment helps to restore the health of grassland ecosystems, increase vegetation coverage and biodiversity, improve the regional ecological environment, and prevent further land degradation [38]. Therefore, it is crucial to strengthen the treatment of saline-alkali land in significant and extremely significant degradation areas to promote ecological restoration and sustainable development in the region.

### 4.3. Analysis of Influencing Factors of GRI in the West Songnen Plain

The spatio-temporal distribution of grassland ecosystem resilience in the western Songnen Plain was the result of joint impacts of geographical location, climate, and human activities. A higher latitude was the main controlling factor for the generally lower resilience of grassland ecosystems in the Songnen Plain. Although the altitude was low, the effective accumulated temperature was low due to the distance from the equator. Because the low temperature and low heat seriously restrict soil microbial activity and delay the decomposition process of soil organic matter, many forage grasses find it difficult to enter the seed maturation stage [61,62,63]. In addition, due to differences in latitude, soils show zonal changes from north to south. The accumulation thickness of organic matter in the soil of the northern Heilongjiang Province is generally thicker than that of the southern Jilin Province [64], which is the main reason for the appearance of high-value GRI clustering areas in the Heilongjiang Province (Figure 5).

Previous studies showed that climate change is the primary contributing factor to soil degradation and vegetation change in northeast China [65,66]. Our study proved that variable climate was also the primary influencing factor in changes in the GRI. Affected by the global El Niño and La Niña phenomenon, the overall trend of temperature and precipitation is on the rise in the study period in the Songnen Plain, and the climate tends to be warm and humid [67]. This trend was conducive to the improvement of vegetation resilience (Figure 7), but regional heterogeneity was significant. Spatially, the MAT and MAP significantly increased precipitation in the north and west of the study area, respectively. From 2000 to 2020, the mean contribution of the MAT and MAP varied between 41.12% and 52.70% (Figure 8). The RCR of climate variables for Songyuan in 2020 was as high as 61% (Figure 8). Changes in the GRI mainly showed a positive relationship with temperature and a negative relationship with precipitation, indicating that temperature is the main factor affecting vegetation restoration in high-latitude and cold regions. This was also confirmed in relevant studies by different research [68,69,70]. In addition, it is possible that the GRI of this paper not only considers the index changes represented by vegetation but also measures the time characteristics of resilience. For example, the increase in temperature may result in an earlier onset of the growing season and a later ending to the growing season [68,71,72], which shortens the recovery time of grassland.

Due to the difficulty in quantifying the livestock carrying capacity that affects grassland resilience in space, we used the GDP and PD as proxies to analyze the impacts of human activities. As the earliest industrial base in China, northeast China has encountered difficulties and crises in its traditional industries due to the transformation of the market economy and global competition, resulting in stagnation in the economy and lower GDP growth rates [73]. In response, the government launched the Northeast Revitalization Strategy to foster economic development, notably introducing the Rural Revitalization Strategy in 2017 to boost rural economies. Despite these efforts, research indicates that the region continues to experience economic contraction and demographic decline [74]. These factors result in small and fluctuating contributions of regional GDP and PD to the resilience of grassland ecosystems. In addition, in recent years, the implementation of a grassland grazing ban and house-feeding policies has led to more grazing grasslands being used as mowing grasslands for feed production in the Songnen grasslands [75]. On the one hand, this has increased the economic income of farmers, and on the other hand, it has become a positive factor in the restoration of grassland ecosystems.

Many studies have attempted to distinguish the relative role of climate and human factors in grassland changes. Previous studies in the northern Songnen Plain and northeast China found that climatic factors predominantly influence changes in vegetation cover and ANPP [34,57], and the increase in precipitation and the implementation of ecological restoration measures are effective factors. Yang et al. put forward the argument that climate change served as the primary driver of grassland degradation in China from 2000 to 2013 [76]. In this study, it was found that the effect of climatic factors on the GRI was significantly higher than that of human activities. The reason might arise from the demarcation criteria and the timeframe employed to delineate grassland areas in this study. Specifically, the delimitation rules utilized in this paper primarily focus on areas where grassland has remained unchanged over the past 22 years, predominantly within the prohibited grazing zones protected by policy measures. Furthermore, since the enactment of grassland protection policies in 2000, the influence of human activities has notably diminished. In addition, research indicated that the implementation of GESAS and the grazing exit plan may have contributed to this effect to a certain extent [77].

Although some research results have been achieved, there are still some deficiencies. The impact of human activities on the GRI is complex. This paper only considers the influence of the GDP and PD. The impact of indicators such as grazing intensity, number of livestock, policy orientation, and grassland management methods on the GRI needs to be considered in future research. In the context of global warming and more extreme climate events, the impact of such events on the resilience of the regional GRI should be considered when studying the dynamics of the GRI, especially for ecologically fragile areas like research areas.

## 5. Conclusions

In this paper, the GRI of the west Songnen Plain was constructed based on the LAI image data, and the dynamic characteristics of grassland resilience and its influence factors from 2000 to 2021 were analyzed. The robustness of the GRI, which considers both vegetation changes and recovery time, has been confirmed through comparison with existing research. The results indicated that the level of grassland resilience in the study area was generally low. The average GRI displayed the characteristics of high-value aggregation in the north and low-value distribution in the south, with the GRI in the Heilongjiang Province having a higher value than those in the Jilin Province. Trend analysis indicated that the areas with an improved GRI accounted for 59.09% of the total grassland area, while there were still some serious degradation areas. Climate change had the most significant impact on the GRI, with the influence of latitude decreasing and that of human activities increasing over the study period. Additionally, we found the effects of dominant factors varied at different scales. The research results contribute to targeted saline-alkali land treatment in severely degraded grassland areas. By combining the findings of this study on the impact of climate change and human activities on the resilience dynamics of grasslands in the west Songnen Plain, more effective measures can be taken for saline-alkali land treatment, promoting ecological restoration and sustainable development in the region.

## Figures and Tables

**Table 1:** Trend classification criteria.

Slope	$\left|Z\right|$	Change Type	Slope	$\left|Z\right|$	Change Type
>0	≥2.58	Extremely significant improvement	0<	≥2.58	Extremely significant degradation
	≥1.96	Significant improvement		≥1.96	Significant degradation
	<1.96	Modest improvement		<1.96	Modest degradation

## Data Availability

Data are contained within the article.
